# Synthesis and Antimicrobial Activities of Some New 1,2,4-Triazole Derivatives

**DOI:** 10.3390/molecules15042427

**Published:** 2010-04-08

**Authors:** Hakan Bektaş, Nesrin Karaali, Deniz Şahin, Ahmet Demirbaş, Şengül Alpay Karaoglu, Neslihan Demirbaş

**Affiliations:** 1 Department of Chemistry, Karadeniz Technical University, 61080 Trabzon, Turkey; 2 Department of Biology, Rize University, 53100 Rize, Turkey

**Keywords:** 1,2,4-Triazole, triazolo[3,4-*b*][1,3]benzoxazole, primary amine, Schiff base, Mannich base

## Abstract

Some novel 4,5-disubstituted-2,4-dihydro-3*H*-1,2,4-triazol-3-one (**3, 6, 8, 9**) derivatives and or 3-(4-methylphenyl)[1,2,4]triazolo[3,4-*b*][1,3]benzoxazole (**5**) were synthesized from the reaction of various ester ethoxycarbonylhydrazones (**1a-e**) with several primary amines. The synthesis of 4-amino-5-(4-chlorophenyl)-2-[(5-mercapto-1,3,4-oxadiazol-2-yl)methyl]-2,4-dihydro-3*H*-1,2,4-triazol-3-one **(13)** was performed starting from 4-Amino-5-(4-chlorophenyl)-2,4-dihydro-3*H*-1,2,4-triazol-3-one (**2**) by four steps; then **13** was converted to the corresponding Schiff base (**14**) by using 4-methoxybenzaldehyde. Finally, two Mannich base derivatives of **14** were obtained by using morpholine or methyl piperazine as amine component. All newly synthesized compounds were screened for their antimicrobial activities and some of which were found to possess good or moderate activities against the test microorganisms.

## 1. Introduction

The synthesis of high nitrogen containing heterocyclic systems has been attracting increasing interest over the past decade because of their utility in various applications, such as propellants, explosives, pyrotechnics and especially chemotherapy [1]. In the medicinal chemistry, azoles are widely used and studied class of antimicrobial agents due to their safety profile and high therapeutic index. Among these, Conazoles are a major class of azole-based drugs such as Itraconazole, Fluconazole, Voriconazole, Ravuconazole *etc.* [2,3,4,5]. Some of other major applications of conazoles are on crop protection. As pharmaceuticals, they are used for the treatment of local and systemic fungal infections, which are important problems in phytopathology and especially in medicine, and they are frequently observed in immune-compromised patients suffering from AIDS or subjected to invasive surgery, anti-cancer therapy or graft receivers. 

Several lines of evidence suggest that the primary target of azoles is the heme protein, which cocatalyzes cytochrome P-450-dependent 14α-demethylation of lanosterol. Inhibition of 14-demethylase leads to depletion of ergosterol and accumulation of sterol precursors, including 14-methylated sterols (lanosterol, 4,14-dimethylzymosterol, and 24-methylenedihydrolanosterol), resulting in the formation of a plasma membrane with altered structure and function. The more recent triazole derivatives, such as fluconazole, itraconazole, and voriconazole owe their antifungal activity at least in part to inhibition of cytochrome P-450-dependent 14α-sterol demethylase [6].

The diseases caused by fungal species cause not only improve the cost of therapy but also may lead mortality. Due to the inadequacy of alone standard antibiotic therapy in certain circumstances, more efforts have been focused on addressing the problem of multidrug-resistant bacteria and the decreasing of costs and consequences the obtained results from this [7,8,9]. Tuberculosis (TB) that is another mortal infection, causes to death with approximately three million patients in the world every year. According to the World Health Organization (WHO), about 30 million people will be infected within next 20 years [10]. Thus, the treatment of infections has become an important and challenging problem because of the increasing number of multi-drug resistant microbial pathogens [11]. In spite of a large number of antibiotics and chemotherapeutics available for medical usage, the increasing resistance made it necessary to continue the search for new antimicrobial substances. Though various molecules designed and synthesized for this aim, the efforts have demonstrated that 1,2,4-triazoles and their derivatives could be considered as possible antimicrobial agents, some of them studied in our laboratories [12,13,14,15,16,17,18,19,20,21,22,23,24]. 

Moreover, synthesis of 1,2,4-triazoles fused to another heterocyclic ring has attracted wide spread attention due to their diverse applications as antibacterial-, antidepressant-, antiviral-, antitumorial- and anti-inflammatory agents, pesticides, herbicides dyes, lubricant and analytical reagents [25,26]. Among these, the commonly known systems are generally triazoles fused to pyridines, pyridazines, pyrimidines, pyrazines and triazines. Although there are not many triazoles fused to thiadiazines or thiadiazoles, a number of them are incorporated into a wide variety of therapeutically important compounds possessing a broad spectrum of biological activities [26,27,28,29].

The condensation reaction of relatively small and linear molecules with suitable reagents is a general method leading to the formation of heterocyclic systems [30]. For this purpose, ester ethoxycarbonylhydrazones (**1**) are suitable precursors; a number of azole derivatives have been obtained in our laboratory starting from compound **1** [12,13,14,15,16,17,18,19,20,21]. Other useful intermediates are the compounds incorporating a hydrazide function in their structures [20,23]. 

**Scheme 1:**
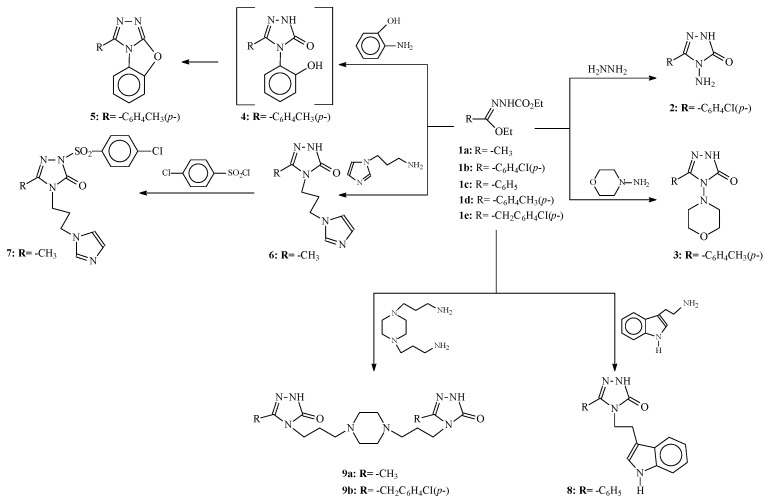
Synthetic pathway for the preparaion of compounds **2–9**.

**Scheme 2:**
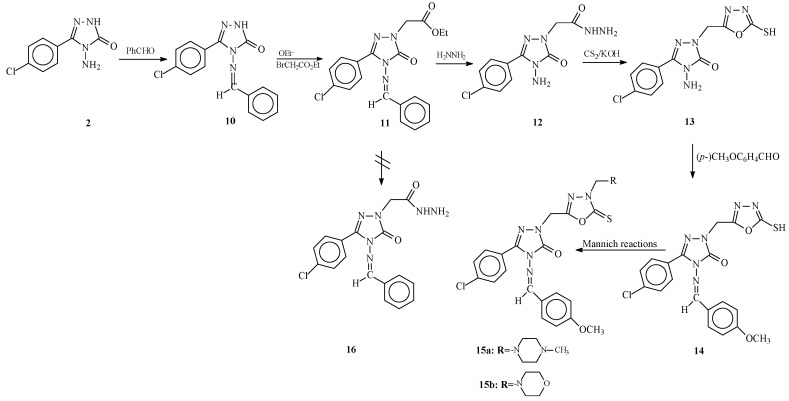
The synthetic routes for compounds **10–15**.

In the present study, as a continuation of our studies on obtaining bioactive molecules, we have performed the synthesis of some new 1,2,4-triazole derivatives and investigation of antimicrobial activities of newly synthesized compounds (Scheme 1, Scheme 2).

## 2. Results and Discussion

The treatment of ester ethoxycarbonylhydrazones (**1a-e)** with various primary amines resulted in the formation of 4,5-disubstitue-2,4-dihydro-3*H*-1,2,4-triazol-3-ones (**3, 6, 8**), while the reaction of **1d** with 2-hydroxyaniline afforded 3-(4-methylphenyl)[1,2,4]triazolo[3,4-*b*][1,3]benzoxazole (**5**). On the other hand, the reaction of **1a,e** with 1,4-di(3-aminopropyl)piperazine produced symmetric bitriazolyl-4-ylpropyl piperazine derivatives (**9a,b**) (Scheme 1). Compounds **3, 5, 6, 8** and **9** exhibited NMR and IR spectra consistent with their assigned structures. In the ^1^H NMR spectrum of **5**, no signal representing an -NH or -OH group was recorded. In addition, compound **5** gave elemental analysis and mass spectral data consistent with the assigned structure (**5**). On the basis of these reported observation, compound **5** was designated as 3-(4-methylphenyl)[1,2,4]triazolo[3,4-*b*][1,3]benzoxazole. 4-Amino-5-(4-chlorophenyl)-2,4-dihydro-3*H*-1,2,4-triazol-3-one (**2**) obtained from the reaction of **1c** with hydrazine hydrate in water at reflux temperature, crystallized from ethanol and gave IR spectroscopic data consistent with literature [31].

The synthesis of ethyl [3-(4-chlorophenyl)-5-oxo-4-{[phenylmethylene]amino}-4,5-dihydro-1*H*-1,2,4-triazol-1-yl]acetate **(11)** was performed by the reaction of compound **10** [27] with ethyl bromoacetate in the presence of sodium ethoxide (Scheme 2). The reaction of compound **11** with hydrazine hydrate in water afforded compound **12** but not **16**. It was reported that the treatment of Schiff bases derived from type **2** compounds might cause to the hydrolysis of alkylidenamino group to free amino group via the attack of hydrazine hydrate (or water) to imine bond at the same time with the attack to exocyclic ester group [23]. The ^1^H NMR spectrum of compound **12** showed complete absence of signals relevant to structure **16**. In addition, compound **12** gave M+1 ion peak in the mass spectrum and good elemental analysis results. 

The treatment of **12** with carbon disulphide in basic media caused to the conversion of hydrazide side chain into 5-mercapto-1,3,4-thiadiazole ring, thus, compound **13** was obtained. It is known that 5-mercapto-1,3,4-oxadiazoles are exist as their mercapto-thioxo tautomeric forms [13,14,15]. As a result of this tautomerism, the IR spectrum of **13** displayed two stretching bands, one of which observed at 2750 cm^-1^ belongs to –SH group, the other recorded at 1164 cm^-1^ represents the existent of -C=S group. Moreover, compound **13** gave satisfactory NMR, mass and elemental analysis data.

The synthesis of compounds **15a,b** was carried out by the Mannich reaction of compound **14** with methyl piperazine (for **15a**) or morpholine (for **15b**) in the presence of formaldehyde solution. Additional signals belonging to methyl piperazine or morpholine moiety and methylene linkage were recorded at the related chemical shif values in the ^1^H and ^13^C NMR spectra of **15a,b**. It was reported that, the compounds having Schiff base structure may exist as *E/Z* geometrical isomers about the–N=CH- double bond [12,19,33,34]. The literature survey revealed that, the compounds containing imine bond are present in higher percentage in dimethyl-*d*_6_ sulfoxide solution in the form of geometrical *E* isomer about –N=CH- double bond [33]. The *Z* isomer can be stabilized in less polar solvents by an intramolecular hydrogen bond. In the ^1^H NMR spectra of compounds **14** and **15a,b**, the -N=CH- signals were observed as two sets due to the existence of *E* and *Z* isomers. 

None of the synthesized compounds showed antimicrobial activity against *Candida tropicalis* (Ct) and *Candida albicans* (Ca). Among the compounds 4,5-disubstitue-2,4-dihydro-3*H*-1,2,4-triazol-3-ones (**3, 6, 8**) and **9a,b**; the compounds **3** and **8** exhibited moderate activities towards *Escherichia coli* (Ec) and *Klebsiella pneumoniae* (Kp) which are containing a morpholine (for **3**) or indol-3-ylethyl moiety (for **8**) in the position 4 of 1,2,4-triazol-3-one ring. Similarly, compound **11** having an imine bond and compound **12** possesing hydrazide function in their structures showed moderate activities against *Enterobacter aerogenes (En), Staphylococcus aureus (Sa), Enterococcus faecalis (Ef) and Bacillus careus (Be).*

Good antimicrobial activities were found for compounds **13** and **14** against the test microorganisms, which are including a 5-mercapto-1,3,4-oxadiazole ring bearing to 1,2,4-triazole nucleus via a methylene linkage. The conversion of the amino group in position 4 of 1,2,4-triazole ring into 4-methoxyphenylenamino group caused no change in the antimicrobial activity for compound **14**. Among the Mannich bases of compound **14**, **15b** displayed good antimicrobial activities against the test microorganisms that contain an additional morpholine moiety beside 1,2,4-triazole and 1,3,4-oxadiazole rings, while other Mannich base, **15a,** that has a methyl piperazine nucleus instead of morpholine, exhibited good or moderate activities towards the test microorganisms except *Escherichia coli (Ec)* and *Klebsiella pneumoniae (Kp).*

## 3. Experimental

### 3.1. General

Melting points were determined on a Büchi B-540 melting point apparatus and are uncorrected. ^1^H NMR and ^13^C NMR spectra were recorded on a Varian-Mercury 200 MHz spectrometer. The IR spectra were measured as potassium bromide pellets using a Perkin-Elmer 1600 series FTIR spectrometer. Mass spectra were obtained at a Quattro LC-MS (ESI, 70 eV) Instrument (except compounds **5, 9a, 9b** and **15a**). Combustion analysis was performed on a Costech Elementel Combution System CHNS-O elemental analyzer. All the chemicals were obtained from Fluka Chemie AG Buchs (Switzerland). Compounds **1a-e, 2** and **10** were prepared by the way reported earlier [30,31,32]. 

### 3.2. General Method for the Synthesis of Compounds ***3, 5, 6, 8, 9a,b***

The corresponding compound **1** (10 mmol) was heated in an oil bath with a suitable primer amine (represented in the scheme 1) (10 mmol for **3,5,6, 8**; 20 mmol for **9a,b**) at reflux temperature (between 90–120 °C) for 2 h. After cooling to room temperature, 3–4 mL of ethyl acetate-petroleum ether mixture was added to the viscous residue. On cooling the mixture in cold, a solid appeared. This crude product was recrystallized from an appropriate solvent to afford the desired product. 

*5-(4-Methylphenyl)-4-morpholin-4-yl-2,4-dihydro-3H-1,2,4-triazol-3-one* **(3**). Recrystallized from ethyl acetate; yield 39%, m.p. 226–227 °C. IR spectrum (KBr), ν, cm^-1^: 3200 (NH), 1699 (C=O), 1508 (C=N). ^1^H NMR spectrum (DMSO-*d*_6_), δ, ppm: 2.35 (s, 3H, CH_3_), 2.94 (brs, 4H, 2CH_2_), 3.81 (brs, 4H, 2CH_2_), 7.29 (d, 2H, arH), 7.45 (d, 2H, arH), 11.94 (s, 1H, NH). ^13^C NMR spectrum (DMSO-*d*_6_), δ, ppm: 20.85 (CH_3_), 51.06 (2CH_2_), 66.15 (2CH_2_), arC: [123.69 (C), 127.25 (2CH), 128.87 (2CH), 139.52 (C)], 144.39 (triazole C3), 153.37 (triazole C5). MS (ESI): *m/z* (%) 283 (M +Na, 58), 261 (M^+^, 10), 230 (42), 218 (11), 208 (74), 140 (100), 118 (14), 112 (21). *Anal.* for: C_13_H_16_N_4_O_2_ Calcd. (%): C, 59.99, H, 6.20, N, 21.52. Found, (%): C, 60.18, H, 6.34, N, 21.23. 

*3-(4-Methylphenyl)*[1,2,4]*triazolo[3,4-b]*[1,3]*benzoxazole* (**5**). Recrystallized from ethanol; yield 70%, m.p. 116–117 °C. IR spectrum (KBr), ν, cm^-1^: 1621 (C=N), 1555 (C=N), 1243 (C-O). ^1^H NMR spectrum (DMSO-*d*_6_), δ, ppm: 2.41 (s, 2H, CH_3_), 7.39–7.47 (m, 4H, arH), 7.76–7.82 (m, 2H, arH), 8.10 (d, 2H, arH, *J =* 8.2 Hz). ^13^C NMR spectrum (DMSO-*d*_6_), δ, ppm: 21.07 (CH_3_), arC:[119.55 (CH), 123.54 (2C), 124.72 (CH), 125.24 (CH), 127.13 (3CH), 129.81 (3CH), 142.08 (2C)], 141.40 (triazole C5), 149.99 (triazole C3). *Anal.* for: C_15_H_13_N_3_O Calcd. (%): C, 71.70, H, 5.21, N, 16.72. Found, (%): C, 71.88, H, 5.27, N, 16.57. 

*4-[3-(1H-Imidazol-1-yl)propyl]-5-methyl-2,4-dihydro-3H-1,2,4-triazol-3-one* (**6**). Recrystallized from ethanol; yield 67%, m.p. 105–106 °C. IR spectrum (KBr), ν, cm^-1^: 3106 (NH), 1696 (C=O), 1592 (C=N). ^1^H NMR spectrum (DMSO-*d*_6_), δ, ppm: 1.97 (t, 2H, CH_2_, *J =* 7.0 Hz), 2.06 (s, 3H, CH_3_), 3.47 (t, 2H, CH_2_, *J =* 7.0 Hz), 3.95 (t, 2H, CH_2_, *J =* 7.0 Hz), 6.87 (s, 1H, arH), 7.17 (s, 1H, arH), 7.65 (s, 1H, arH), 11.43 (s, 1H, NH). ^13^C NMR spectrum (DMSO-*d*_6_), δ, ppm: 15.93 (CH_3_), 34.73 (CH_2_), 42.09 (CH_2_), 48.03 (CH_2_), arC: [123.91 (CH), 133.00 (CH), 141.94 (CH)], 148.99 (triazole C3), 159.71 (triazole C5); MS (ESI): *m/z* (%) 230 (M+Na, 21), 208 (M+1, 15), 140 (100), 112 (22). *Anal.* for: C_9_H_13_N_5_O Calcd. (%): C, 52.16, H, 6.32, N, 33.79. Found, (%): C, 52.28, H, 6.49, N, 33.57.

*4-[2-(1H-Indol-3-yl)ethyl]-5-phenyl-2,4-dihydro-3H-1,2,4-triazol-3-one* (**8**). Recrystallized from ethanol; yield 37%, m.p. 205–206 °C. IR spectrum (KBr), ν, cm^-1^: 3397 (NH), 3379 (NH), 1708 (C=O), 1618 (C=N). ^1^H NMR spectrum (DMSO-*d*_6_), δ, ppm: 2.88 (t, 2H, CH_2,_ *J =* 7.2 Hz), 3.90 (t, 2H, CH_2_, *J =* 7.2 Hz), 6.89 (t, 1H, arH, *J =* 7.8 Hz), 7.00-7.07 (m, 2H, arH), 7.19 (d, 1H, arH, *J =* 8.2 Hz), 7.30 (d, 1H, arH, 8.0 Hz), 7.42-7.52 (m, 6H, arH), 10.84 (s, 1H, NH), 11.93 (s, 1H, NH). ^13^C NMR spectrum (DMSO-*d*_6_), δ, ppm: 24.05 (CH_2_), 41.81 (CH_2_), ar C:[109.83 (C), 111.24 (C), 117.63 (C), 118.21 (C), 120.85 (C), 122.87 (C), 126.76 (C), 127.31 (C), 127.80 (2C), 128.63 (2C), 129.84 (C), 136.01 (C)], 150.99 (triazole C3), 155.02 (triazole C5). MS (ESI): *m/z* (%) 327 (M+Na, 100), 324 (16), 305 (M+, 24), 189 (25), 188 (25), 153 (22), 148 (34), 144 (94), 121 (24). *Anal.* for: C_18_H_16_N_4_O Calcd. (%): C, 71.04, H, 5.30, N, 18.41. Found, (%): 71, 18, H, 5.1, N, 18.27. 

5-Methyl-4-(3-{4-[3-(3–methyl-5-oxo-1,5-dihydro-4H-1,2,4–triazol–4-yl)propyl] piperazin-1-yl}propyl)-2,4-dihydro-3H-1,2,4-triazol-3-one (**9a**). Recrystallized from ethanol; yield 59%, m.p. 246–248 °C. IR spectrum (KBr), ν ,cm^-1^: 3177 (NH), 1707 (C=O), 1586 (C=N). ^1^H NMR spectrum (DMSO-d_6_), δ, ppm: 1.74 (brs, 4H, 2CH_2_), 2.15-2.24 (brs, 18H, 6CH_2_+2CH_3_), 3.40 (brs, 4H, 2CH_2_), 11.31 (s, 2H, 2NH). ^13^C NMR spectrum (DMSO-d_6_), δ, ppm: 11.31 (CH_3_), 25.15 (2CH_2_), 52.54 (6CH_2_), 54.51 (2CH_2_), 144.51 (triazole C3), 154.96 (triazole C5). Anal. for: C_16_H_18_N_8_O_2_ Calcd. (%): C, 49.99, H,7.19, N, 33.31. Found, (%): C, 50.18, H, 6.95, N, 33.17. 

5-(4-Chlorobenzyl)-4-(3-{4-[3-(3-methyl-5-oxo-1,5-dihydro-4H-1,2,4-triazol-4-yl)propyl] piperazin-1-yl}propyl)-2,4-dihydro-3H-1,2,4-triazol-3-one (**9b**). Recrystallized from ethanol/ water [1:1]); yield 81%, m.p. 214–216 °C. IR spectrum (KBr), ν ,cm^-1^: 3183 (NH), 1698 (C=O), 1569 (C=N).^1^H NMR spectrum (DMSO-d_6_), δ, ppm: 1.51 (brs, 4H, 2CH_2_), 2.18 (brs, 12H, 6CH_2_), 3.43 (brs, 4H, 2CH_2_), 3.97 (brs, 4H, 2CH_2_), 7.30–7.39 (m, 8H, arH), 11.55 (brs, 2H, 2NH). ^13^C NMR spectrum (DMSO-d_6_), δ, ppm: 24.82 (2CH_2_), 30.53 (2CH_2_), 52.44 (6CH_2_), 54.30 (2CH_2_), arC: [128.50 (4CH), 130.34 (4CH), 131.54 (2C), 134.55 (2C)], 145.97 (2 triazole C-5), 155.01 (2 triazole C-3). Anal. for: C_28_H_34_CI_2_N_8_O_2_ Calcd. (%): C, 56.02 H, 5.42 N, 20.10. Found, (%): C, 56.24 H, 5.80 N, 19.91.

*2-[(4-Chlorophenyl)sulfonyl]-4-[2-(1H-indol-3-yl)ethyl]-5-methyl-2,4-dihydro-3H-1,2,4-triazol-3-one* (**7**). Compound **6** (10 mmol) was refluxed with 1 equivalent of sodium in dichloromethane for 8 hours. Then, 4-chlorobenzenesulphonyl chloride (10 mmol) was added and refluxed for an additional 8 hours. After evaporating the solvent under reduced pressure, a solid appeared. This was recrystallized form ethanol to afford compound **7**. Yield 48%, m.p. 147–149 °C. IR spectrum (KBr), ν, cm^-1^: 1730 (C=O), 1603 (C=N). ^1^H NMR spectrum (DMSO-*d*_6_), δ, ppm: 2.14 (t, 2H, CH_2_, *J =* 6.4 Hz), 2.21 (s, 3H, CH_3_), 3.53 (t, 2H, CH_2_, *J =* 6.6 Hz), 4.16 (t, 2H, CH_2_, *J =* 6.6 Hz), 7.38 (d, 1H, arH, *J =* 8.2 Hz), 7.60 (d, 2H, arH, *J =* 8.6 Hz), 7.80–7.73 (m, 2H, arH), 7.95 (d, 1H, arH, *J =* 8.6 Hz), 8.95 (s, 1H, arH). ^13^C NMR spectrum (DMSO-*d*_6_), δ, ppm: 11.51 (CH_3_), 28.29 (CH_2_), 36.86-40.58 (DMSO-*d*_6_ + CH_2_), 45.57 (CH_2_), arC: [125.74 (2CH), 127.54 (2CH), 128.38 (CH), 129.28 (CH), 129.94 (CH), 135.27 (C), 140.02 (C)], 148.86 (triazole C3), 150.95 (triazole C5). MS (ESI): *m/z* (%) 384 (M+2, 26), 382 (M^+^, 66), 316 (64), 314 (100), 175 (13). *Anal.* for: C_15_H_16_CIN_5_O_3_S Calcd. (%): C, 47.18 H, 4.22 N, 18.34. Found, (%): C, 47.37, H, 4.33 N: 18.28. 

*Ethyl [3-(4-chlorophenyl)-5-oxo-4-{[phenylmethylene]amino}-4,5-dihydro-1H-1,2,4-triazol-1-yl]acetate* (**11**). The corresponding compound **10** (10 mmol) was refluxed with 1 equivalent of sodium in absolute ethanol for 2 hours. Then, ethyl bromoacetate (10 mmol) was added and refluxed for an additional 8 hours. After evaporating the solvent under reduced pressure, a solid appeared. This was recrystallized from ethanol/water (1:2) to afford compound **11**. Yield 95%, m.p. 131–132 °C. IR spectrum (KBr), ν, cm^-1^: 1742 (C=O), 1714 (C=O), 1579 (C=N), 1230 (C-O). ^1^H NMR spectrum (DMSO-*d*_6_), δ, ppm: 1.18–1.26 (t, 3H, CH_3,_ *J =* 7.4 Hz ), 4.13-4.23 (q, 2H, CH_2_CH_3_, *J =* 7.4 Hz), 4.77 (s, 2H, NCH_2_), 7.48–7.65 (m, 5H, ar-H), 7.81–7.95 (m, 4H, ar-H), 9.62 (s, N=CH).^13^C NMR spectrum (DMSO-*d*_6_), δ, ppm: 15.81 (CH_3_), 48.65 (CH_2_), 63.18 (CH_2_), arC: [126.51 (C), 129.91 (2C), 130.65 (2C), 130.92 (2C), 131.59 (C), 133.77 (C), 134.75 (C), 137.14 (C), 144.70 (C)], 144.7 (N=CH), 151.54 (triazole C-3), 159.24 (triazole C-5), 169.28 (C=O). MS (ESI): *m/z* (%) 385.82 (M+1, 26), 272 (80). *Anal.* Calcd. (%) for: C_19_H_17_N_4_CIO_3_: C, 59.30, H, 4.45, N, 14.56. Found, (%): C, 59.20, H, 4.42, N,14.55. 

*2-[4-amino-3-(4-chlorophenyl)-5-oxo-4,5-dihydro-1H-1,2,4-triazol-1-yl]aceto hydrazide* (**12**). A solution of the corresponding compound **11** (10 mmol) in *n*-butanol was refluxed with hydrazine hydrate (25 mmol) for 4 hours. After cooling it to room temperature, a white solid appeared. This was recrystallized from ethanol to afford the desired product. Yield 98%, m.p. 215–218 °C. IR spectrum (KBr), ν, cm^-1^: 3302–3213 (NH+2NH_2_), 1717 (C=O), 1668 (C=O), 1577 (C=N). ^1^H NMR spectrum (DMSO-*d*_6_), δ, ppm: 4.34 (s, 2H, NCH_2_), 4.46 (s, 2H, NHNH_2_), 5.55 (s, 2H, NH_2_), 7.52-7.65 (m, 2H, ar-H), 7.82-8.05 (m, 2H, ar-H), 9.27 (s, NHNH_2_). ^13^C NMR spectrum (DMSO-*d*_6_), δ, ppm: 48.69 (CH_2_), arC: [126.82 (C), 127.38 (C), 133.82 (C), 134.93 (C), 138.47(C), 138.97 (C)], 151.79 (triazole C-3), 158.55 (triazole C-5), 167.45 (C=O). MS (ESI): *m/z* (%) 283.69 (M+1, 32), 305 (100), 273 (34), 229(48). *Anal.* Calcd. (%) for: C_10_H_11_N_6_ClO_2_: C, 42.49, H, 3.92, N, 29.73. Found, (%): C, 42.50, H, 3.90, N,29.65. 

*4-Amino-5-(4-chlorophenyl)-2-[(5-mercapto-1,3,4-oxadiazol-2-yl)methyl]-2,4-dihydro-3H-1,2,4-triazol-3-one* (**13**). Compound **12** (10 mmol) and CS_2_ (6.0 mL, 10 mol) were added to a solution of KOH (0.56 g, 10 mol) in 50 mL H_2_O and 50 mL ethanol. The reaction mixture was refluxed for 3 h. After evaporating in reduced pressure to dryness, a solid was obtained. This was dissolved in 300 mL H_2_O and acidified with conc. HCl. The precipitate was filtered off, washed with H_2_O and recrystallized from ethanol to afford the desired compound.Yield 89%, m.p. 220–221 °C. IR spectrum (KBr), ν, cm^-1^: 3323–3203 (NH_2_), 2750 (SH), 1679(C=O), 1518, 1492 (C=N), 1164 (C=S).^1^H NMR (DMSO-*d*_6_), δ, ppm: 5.18 (s, 2H, CH_2_), 5.63 (bs, 2H, NH_2_), 7.55–7.59 (d, 2H, arH, *J =* 8.4 Hz), 7.98–8.02 (d, 2H, arH, *J =* 8.4 Hz), 14.65 (bs, 1H, SH).^13^C NMR spectrum (DMSO-*d*_6_), δ, ppm: 38.085–40.172 (DMSO-*d*_6_ + CH_2_), arC: [125.14 (2C), 128.45 (C), 130.80 (2C), 134.12 (C)], 152.70 (triazole C-3), 155.12 (triazole C-5), 160.12 (oxadiazole C-2), 174.12 (oxadiazole C-5). MS (ESI): *m/z* (%) 363 (100), 347 (M+Na, 12), 325.74 (M+1, 36), 223 (54), 123 (84). *Anal.* Calcd. (%) for: C_11_H_9_N_6_ClO_2_S: C, 40.86, H, 2.79, N, 25.88. Found, (%): C, 40.62, H, 2.80, N,25.80. 

*5-(4-Chlorophenyl)-2-[(5-mercapto-1,3,4-oxadiazol-2-yl)methyl]-4-[(4-methoxyben-zylidene)amino]-2,4-dihydro-3H-1,2,4-triazol-3-one* (**14**). The mixture of compound **13** (10 mmol) and anisaldehyde (10 mmol) in absolute ethanol was refluxed for 5h. On cooling it to room temperature, a solid appeared. This crude product was recryristallized from ethanol: water (1:1) to obtain compound **14**. Yield 71%, m.p. 229–230 °C. IR spectrum (KBr), ν, cm^-1^: 2747 (SH), 1705 (C=O), 1628, 1605, 1566 (3C=N), 1170 (C=S). ^1^H NMR spectrum (DMSO-*d*_6_), δ, ppm: 3.84 (s, 3H, OCH_3_), 5.28 (s, 2H, NCH_2_), 7.05–7.09 (d, 2H, ar-H, *J =* 8.6 Hz), 7.58–7.62 (d, 2H, ar-H, *J =* 8.2 Hz), 7.66–7.81(d, 2H, ar-H, *J* =8.6 Hz), 7.90-7.94 (d, 2H, ar-H, *J* =8.2 Hz), 9.49 and 9.63 (s, 1H, -N=CH, *E/Z* geometrical izomers), 14.74 (bs, 1H, SH). ^13^C NMR spectrum (DMSO-*d*_6_), δ, ppm: 38.085-40.17 (DMSO-*d*_6_ + CH_2_), 55.31 (OCH_3_), arC: [114.43 (2C), 124.35 (C), 125.15 (2C), 127.96 (C), 128.66 (2C), 129.86 (2C), 135.24 (C), 162.19 (C)], 143.21 (triazole C-3), 149.21 (triazole C-5), 157.73 (C=N), 158.47 (oxadiazole C-2), 177.96 (oxadiazole C-5). MS (ESI): *m/z* (%) 443.85 (M+1) (42), 305 (80), 131 (56), 123 (73). *Anal.* Calcd. (%) for: C_19_H_15_N_6_ClO_3_S: C, 51.53, H, 3.41, N, 18.98. Found, (%): C, 51.48, H, 3.45, N,18.90. 

### 3.3. General Method for the synthesis of compounds ***15a, 15b***

To the solution of corresponding compound **14** (10 mmol) in dichloromethane, formaldehyde (37%, 1.55 mL) and methyl piperazine (for **15a**) or morpholine (for **15b**) (10 mmol) were added and the mixture was stirred at room temperature for 3 h. After removing the solvent under reduce pressure, a solid was obtained. This crude product was treated with water, filtered off and recrystallized from ethyl acetate/petroleum ether (1:2) to yield the title compounds. 

*4-[(4-Methoxybenzylidene)amino]-5-(4-chlorophenyl)-2-({4-[(4-methylpiperazin-1-yl)methyl]-5-thioxo-4,5-dihydro-1,3,4-oxadiazol-2-yl}methyl)-2,4-dihydro-3H-1,2,4-triazol-3-one* (**15a**). Yield 75%, m.p. 165–167 °C. IR spectrum (KBr), ν, cm^-1^: 2985 (CH), 1715 (C=O), 1616,1510 (C=N), 1330 (C=S). ^1^H NMR spectrum (DMSO-*d*_6_), δ, ppm: 2.29 (s, 3H, NCH_3_), 2.52 (bs, 4H, 2CH_2_), 2.78 (bs, 4H, 2CH_2_), 3.84 (s, 3H, OCH_3_), 4.98 (s, 2H, CH_2_), 5.21 (s,2H, NCH_2_), 7.06–7.10 (d, 2H, ar-H, *J =* 8.6 Hz), 7.60–7.64 (d, 2H, ar-H, *j* =8.2 Hz), 7.78–7.83 (d, 2H, ar-H, *J =* 8.6 Hz), 7.91–7.95 (d, 2H, ar-H, *J =* 8.2 Hz), 9.50,9.64 (s, 1H, -N=CH, *E/Z* geometrical izomers). ^13^C NMR (DMSO-*d*_6_), δ, ppm: 44.58 (NCH_3_), 48.68 (2C, 2CH_2_), 52.30 (2C, 2CH_2_), 53.65 (NCH_2_), 55.38 (OCH_3_), 69.39 (NCH_2_N), arC: [114.52 (2C), 124.58 (C), 125.22 (C), 128.71 (2C), 129.04 (2C), 129.95 (2C), 135.21 (C), 162.24 (C)], 143.10 (triazole C-3), 149.37 (triazole C-5), 156.97 (C=N), 157.88 (oxadiazole C-2), 164.22 (oxadiazole C-5). *Anal.* Calcd. (%) for: C_25_H_27_N_8_ClO_3_S: C, 54.10, H, 4.90, N, 20.19. Found, (%): C, 54.11, H, 4.88, N, 20.15. 

4-[(4-Methoxybenzylidene)amino]-5-(4-chlorophenyl)-2-{[4-(morpholin-4-ylmethyl)-5-thioxo-4,5-

*dihydro-1,3,4-oxadiazol-2-yl}methyl)-2,4-dihydro-3H-1,2,4-triazol-3-one* (**15b**). Yield 78%, m.p. 175–176 °C. IR spectrum (KBr), ν, cm^1^: 1712 (C=O), 1606, 1515 (2C=N), 1327 (C=S). ^1^HNMR spectrum (DMSO-*d*_6_), δ, ppm: 2.69 (t, 4H, 2CH_2_), 3.56 (t, 4H, 2CH_2_), 3.84 (s, 3H, OCH_3_), 4.97 (s, 2H, CH_2_), 5.30 (s, 2H, NCH_2_), 7.05–7.10 (d, 2H, ar-H, *J* =8.8 Hz), 7.60-7.64 (d, 2H, ar-H, *J =* 8.6 Hz), 7.78–7.82 (d, 2H, ar-H, *J* = 8.6 Hz), 7.90–7.94 (d, 2H, ar-H, *J =* 8.8 Hz), 9.49, 9.63 (s,1H, N=CH, *E/Z* geometrical izomers).^13^CNMR spectrum (DMSO-*d*_6_), δ, ppm: 50.5 (2C, 2CH_2_), 56.137 (OCH_3_), 64.16 (NCH_2_), 66.65 (2C, 2CH_2_), 70.43 (NCH_2_N), arC: [115.28 (2C), 125.29 (C), 125.95 (C), 129.48 (2C), 130.49 (2C), 130.72 (2C), 136.07 (C), 163.03 (C)], 144.08 (triazole C-3), 150.18 (triazole C-5), 157.61 (C=N), 158.55 (oxadiazole C-2), 178.68 (oxadiazole C-5). MS (ESI): *m/z* (%) 542 (M^+^, 20), 508 (32), 292 (30), 215 (83), 210 (94), 153 (100). *Anal.* Calcd. (%) for: C_24_H_24_N_7_ClO_4_S: C,53.18, H, 4.46, N, 18.09. Found, (%): C, 53.15, H, 4.45, N,18.12.

### 3.4. Antimicrobial activity assessment

All test microorganisms were obtained from the Hifzissihha Institute of Refik Saydam (Ankara, Turkey) and were as follows: *Escherichia coli* ATCC 35218, *Klebsiella pneumoniae* ATCC 13883, *Yersinia pseudotuberculosis* ATCC 911, *Enterobacter aerogenes* ATCC 13048, *Pseudomonas aeruginosa* ATCC 10145, *Staphylococcus aureus* ATCC 25923, *Enterococcus faecalis* ATCC 29212, *Bacillus cereus* 709 Roma, *Candida tropicalis* ATCC 13803, Candida glabrata 66032 and *Candida albicans* ATCC 60193. All the newly synthesized compounds were weighed and dissolved in dimethylsulphoxide to prepare extract stock solution of 10.000 microgram/milliliter (μg/mL). 

The antimicrobial effects of the substances were tested quantitatively in respective broth media by using double dilution and the minimal inhibition concentration (MIC) values (µg/mL) were determined [35,36]. The antibacterial and antifungal assays were performed in Mueller-Hinton broth (MH) (Difco, Detroit, MI) at pH 7.3 and buffered Yeast Nitrogen Base (Difco, Detroit, MI) at pH 7.0, respectively. The MIC was defined as the lowest concentration that showed no growth. Ampicillin (10 μg) was used as standard antibacterial and antifungal drugs, respectively. Dimethylsulphoxide with dilution of 1:10 was used as solvent control. The results are shown in Table 1.

## 4. Conclusion

This study reports the successful synthesis of some new 1,2,4-triazol-3-one derivatives, one of them into the corresponding Shiff and Mannich bases. The antimicrobial screening studies were also performed in the study. 1,2,4-Triazole nucleus is one of the active components present in many standard drugs and it is known to increase the pharmacological activity of the molecules. The presence of *N*-methylpiperazine or morpholine moiety is also instrumental in contributing to the net biological activity of a system.

Also we already reported antimicrobial activities of some biheterocyclic triazole derivatives incorporating indole, imidazole, 1,3,4-oxadiazole and piperazine moieties. Hence herein we combined all these potential units, namely 1,2,4-triazole and 1,3,4-oxadiazole, imidazole, indole, morpholine, piperazine or methyl piperazine ring. 

The antimicrobial screening suggests that among the newly synthesized compounds, the compounds **3** and **8** exhibited moderate activities towards *Escherichia coli* (Ec) and *Klebsiella pneumoniae* (Kp), similarly, compounds **11** and **12** showed moderate activities against *Enterobacter aerogenes (En), Staphylococcus aureus (Sa), Enterococcus faecalis (Ef) and Bacillus careus (Be)*; while good antimicrobial activities were found for compounds **13** and **14** against the test microorganisms. Also the Mannich bases **15a,b** displayed good or moderate antimicrobial activities against the test microorganisms. On the other hand, none of the synthesized compounds showed antimicrobial activity against *Candida tropicalis* (Ct) and *Candida albicans* (Ca).

## Figures and Tables

**Table 1:** Antimicrobial activity of the synthesized compounds (µg/mL).

Compound No.	Microorganisms and inhibition zone (mm)									
	Ec	Kp	Yp	En	Pa	Sa	Ef	Bc	Ca	Ct
**3**	250	250	>500	>500	>500	>500	>500	>500	>500	>500
**5**	>500	>500	>500	>500	>500	>500	>500	>500	>500	>500
**6**	>500	>500	>500	>500	>500	>500	>500	>500	>500	>500
**7**	>500	>500	>500	>500	>500	>500	>500	>500	>500	>500
**8**	250	250	>500	>500	>500	>500	>500	>500	>500	>500
**9a**	>500	>500	>500	>500	>500	>500	>500	>500	>500	>500
**9b**	>500	>500	>500	>500	>500	>500	>500	>500	>500	>500
**11**	>500	>500	>500	>250	>500	250	250	>250	>500	>500
**12**	>500	>500	>500	62.5	>500	125	125	62.5	>500	>500
**13**	<1.95	<1.95	<1.95	<1.95	<1.95	<1.95	<1.95	<1.95	>500	>500
**14**	<1.95	<1.95	<1.95	<1.95	<1.95	<1.95	<1.95	<1.95	>500	>500
**15a**	>500	500	250	7.81	250	15.63	125	1.95	>500	>500
**15 b**	<1.95	<1.95	<1.95	<1.95	<1.95	<1.95	<1.95	<1.95	>500	>500
**Amp.**	10	>128	18	>128	18	35	10	15	>500	>500
**Flu.**									<1	8

Ec: Escherichia coli ATCC 25922, Yp: Yersinia pseudotuberculosis ATCC 911, Pa: Pseudomonas aeruginosa ATCC 27853, Ef: Enterococcus faecalis ATCC 29212, Sa: Staphylococcus aureus ATCC 25923, Kp: Klebsiella pneumoniae ATCC 13883, En: Enterobacter aerogenes ATCC 13048; Bc: Bacillus cereus 702 Roma, Ct: Candida tropicalis ATCC 13803, Ca: Candida albicans ATCC 60193, Amp: Ampicillin, Flu: Fluconazole.
